# Editorial: Fungal cell wall proteins with functions in cell wall biogenesis, cell wall signaling and interactions with host

**DOI:** 10.3389/fcimb.2022.1064386

**Published:** 2022-10-26

**Authors:** Justyna Karkowska-Kuleta, Abhiram Maddi

**Affiliations:** ^1^ Department of Comparative Biochemistry and Bioanalytics, Faculty of Biochemistry, Biophysics and Biotechnology, Jagiellonian University, Krakow, Poland; ^2^ Division of Periodontics, Department of Stomatology, Medical University of South Carolina, James Edwards College of Dental Medicine, Charleston, SC, United States

**Keywords:** pathogenic fungi, fungal cell wall, cell wall proteins, cell signaling, antifungal drug resistance, surface adhesins, infections, virulence

The fungal kingdom comprises a broad diversity of organisms, and although life on Earth would not be possible without them, some fungal species might be dangerous pathogens threatening human health and causing different types of infections, including superficial mycoses and invasive fungal infections that are difficult to treat and associated with high rates of morbidity and mortality. Individuals with congenital or acquired disorders of the immune system, after cancer therapy, surgery, antibiotic treatment, or a prolonged hospital stay, are particularly prone to fungal infections (Pfaller et al., 2019; Pfaller et al., 2020). Most often, these are caused by fungi from the genus *Candida*, *Aspergillus*, and *Cryptococcus*, which are widespread throughout the world, but endemic fungal species of medical importance also require particular attention (Malcolm and Chin-Hong, 2013). Another problem, in addition to human infections, is the abundant occurrence of fungal plant pathogens, which cause severe damage to crops, food contamination, and significantly affect the agriculture industry (Doehlemann et al., 2017).

As fungal infections are a growing global problem, studying the molecular mechanisms underlying host-pathogen interactions is particularly important to develop effective methods of preventing and diagnosing fungal infections. Furthermore, there is a need for new antifungal therapies, especially because of the rapid emergence of drug resistance to the currently available antifungals. The fungal cell wall is the part of the cell that differentiates the pathogens from the human host and provides initial and continuing contact of the fungi with the host proteins and tissues during infection. It is a complex structure that undergoes dynamic changes to ensure that the pathogenic microbe adapts to the challenging conditions in the host organism (Ibe and Munro, 2021). The cell wall of fungi consists of linear and branched polysaccharides, a variety of proteins, some of which are covalently bound to the cell wall and others are more loosely attached, as well as small amounts of lipids and other molecules (Klis et al., 2010). All these surface-exposed components could play an essential role in both the initiation and establishment of the fungal infection (Free, 2013).

The complexity of the fungal cell wall structure is reflected in the multifaceted relationships between pathogens and the host, and the cell wall molecules might be a promising target for new antifungal approaches. Not only those proteins that are displayed at the very surface of fungal cells and directly involved in the interactions with host, in the adhesion process and cell signaling related to cell wall integrity and morphology, may play an important role in the virulence of fungi, but also the involvement of numerous enzymatic proteins in the synthesis and remodeling of other cell wall components is crucial for fungal pathogenesis. In this Research Topic, studies concerning the role of different proteins in the biogenesis of the fungal cell wall and interactions with the host were collected. One of the important groups of cell wall proteins is adhesins responsible for the attachment of fungal cells to the biotic and abiotic surfaces, binding of host proteins, cell-cell interactions, and biofilm formation. In the case of *Candida albicans*, the most common fungal pathogen in humans, several surface-displayed adhesive proteins have been identified to date and their role in pathogenesis of candidiasis has been frequently demonstrated (Butler et al., 2009; Hoyer and Cota, 2016). The production of antibodies against Gln-Pro-rich region of protein Hwp1 (hyphal wall protein 1), which is the major cell wall protein present at the surface of *C. albicans* hyphae and is found also on *C. dubliniensis* filamentous forms, was described in the work by Oh et al. (2022). It could be particularly beneficial for the proper diagnosis of candidial infections, and also for detailed studies of the interactions of fungal pathogens with the human host. Furthermore, Hwp1 protein of *C. albicans* is crucial for adhesion to host cells and for biofilm formation, also through colocalization and interactions with important adhesins form agglutinin-like sequence protein family, namely Als1p and Als3p.

In addition to surface adhesins, other proteins related to cell wall biosynthesis are important in *C. albicans* virulence. Using a carefully controlled set of *C. albicans* isogenic mutants defective in genes related to the cell wall construction and maintenance, Sem et al. (2016) demonstrated, that not simply the cell wall composition and the total level of particular wall polysaccharides, but mainly the dynamic architecture of this structure is important for the recognition of fungi by the host immune cells and the fitness of pathogens to the mammalian gastrointestinal (GI) tract. Importantly, surface exposition of β-glucan recognized by the Dectin-1 receptor, dependent on the activity of different enzymes involved in cell wall production, was highly correlated with a loss of competitive fitness of *C. albicans* in the environment of murine GI tract. In further studies by Huang et al. (2020), the derivative of 2-aminonicotinamide−compound 11g−was described as an agent with function in unmasking cell wall exposed β-glucan, thus enhancing the response of host immune cells to fungal pathogens. In the research article by Lv et al. (2020) the studies on mycoparasite *Clonostachys rosea* were demonstrated and the important role of cell wall biogenesis protein phosphatase CrSsd1 in fungal conidiation, hyphal growth, response to osmotic stress, cell wall integrity and mycoparasitism was proved. In the review by Ortiz-Ramírez et al. (2022) the fungal resistance to stress conditions, described for *Sporothrix schenckii* and *Sporothrix globose*, was correlated with the activity of an enzyme GlcN-6-P synthase (L-glutamine:D-fructose-6-phosphate amidotransferase) indicating some specific cellular compensatory responses to the damage of fungal cell wall.

Cell wall-related proteins have numerous important functions and represent ideal targets for novel diagnostic methods, vaccines and new antifungal drugs. Even though some functions have been explored already, many questions still remain to be answered to get a comprehensive outlook of the participation of individual components of the fungal cell wall, as well as this structure as a whole, in contact with the host.

## Author contributions

All authors listed have made a substantial, direct, and intellectual contribution to the work and approved it for publication.

## Conflict of interest

The authors declare that the research was conducted in the absence of any commercial or financial relationships that could be construed as a potential conflict of interest.

## Publisher’s note

All claims expressed in this article are solely those of the authors and do not necessarily represent those of their affiliated organizations, or those of the publisher, the editors and the reviewers. Any product that may be evaluated in this article, or claim that may be made by its manufacturer, is not guaranteed or endorsed by the publisher.
